# Modulation of Phytochemical Pathways and Antioxidant Activity in Peppermint by Salicylic Acid and GR24: A Molecular Approach

**DOI:** 10.3390/cells13161360

**Published:** 2024-08-15

**Authors:** Parisa Jariani, Manijeh Sabokdast, Taraneh Karami Moghadam, Farzaneh Nabati, Beata Dedicova

**Affiliations:** 1 Department of Agriculture and Plant Breeding, Faculty of Agriculture and Natural Resources, University of Tehran, P.O. Box 4111, Karaj 77871-31587, Iran; parisa.jariani@ut.ac.ir (P.J.); karami.taraneh.m@ut.ac.ir (T.K.M.); 2 Medicinal Plants Research Center, Institute of Medicinal Plants, The Academic Center for Education, Culture and Research (ACECR), Karaj 33651-66571, Iran; nabati@imp.ac.ir; 3 Department of Plant Breeding, Swedish University of Agricultural Sciences (SLU) Alnarp, Sundsvägen 10, P.O. Box 190, SE-234 22 Lomma, Sweden

**Keywords:** *Mentha piperita* L., oxidative stress, salicylic acid, strigolactone

## Abstract

This study uncovers the potential of salicylic acid (SA) and synthetic Strigolactone (GR24) in enhancing menthol biosynthesis and antioxidant defense mechanisms in *Mentha piperita* L. Our comprehensive analysis, which included a series of controlled experiments and data analysis of the effects of these phytohormones on enzymatic antioxidants catalase (CAT) and ascorbate peroxidase (APX) and non-enzymatic antioxidants, including carotenoids and proline, revealed promising results. The study also examined their impact on lipid peroxidation, hydrogen peroxide levels, and the expression of genes critical to menthol and menthofuran synthesis. The results indicated that SA and GR24 significantly increased menthol production and reduced the levels of menthofuran and pulegone, suggesting upregulation in the plant’s innate defense systems. Furthermore, the activities of CAT and APX were elevated, reflecting a strengthened antioxidant response. Interestingly, the menthofuran synthase (*MFS*) was higher in the control group. At the same time, pulegone reductase (*PR*) genes and menthol dehydrogenase (*MDH*) gene expression were upregulated, highlighting the protective effects of SA and GR24. These findings underscore the potential of SA and GR24 to serve as effective bio-stimulants, improving the quality and resilience of peppermint plants and thereby contributing to eco-friendly agricultural practices in pollution-stressed environments.

## 1. Introduction

In the quest for sustainable agricultural practices, bio-stimulants have emerged as crucial agents in enhancing plant resilience and productivity [1,2]. These substances, which include natural and synthetic compounds, play a significant role in fortifying plant defenses against various stressors, thereby preserving crop quality and yield [3]. Central to the effectiveness of bio-stimulants is their ability to modulate phytohormones—key endogenous messengers essential for plant development, growth, and stress response [4,5]. Phytohormones regulate a wide range of cellular functions and stress responses, such as strengthening cell walls, maintaining pH balance, promoting root hair growth, facilitating ion transport, synthesizing chlorophyll, and structuring leaves [6,7].

These functions collectively contribute to enhanced plant stress tolerance, resulting in improved seed germination, increased seedling vigor, more efficient leaves, a robust root system, and reduced oxidative damage [8,9]. This study specifically explores the molecular and biochemical pathways triggered by SA and the synthetic strigolactone analog GR24 in *Mentha piperita* L., commonly known as peppermint [10,11,12]. Peppermint, a member of the Lamiaceae family, is renowned for its medicinal and aromatic properties, making it a valuable crop in the food, cosmetic, pharmaceutical, and essential oil sectors [13,14].

As a significant producer of EO, peppermint contributes over a million kilograms annually to the global market [15,16]. Its essential oil primarily consists of menthol and menthone, which are key components in various applications [17]. This study aims to elucidate how SA and GR24 influence the growth and stress responses of peppermint, potentially enhancing its productivity and quality.

This comprehensive approach not only underscores the importance of bio-stimulants in sustainable agriculture but also highlights the specific benefits of phytohormone modulation in improving plant resilience and productivity.

This paper aims to elucidate the intricate biosynthetic processes of menthol, a key component of peppermint EO, and explore the plant’s antioxidative defense mechanisms. This understanding is crucial not only for the scientific community but also for industries that rely on peppermint’s EO. *Mentha piperita* L., commonly known as peppermint, is a perennial herb highly regarded for its aromatic and medicinal properties. The plant’s EO, particularly menthol, holds significant commercial value in the pharmaceutical, cosmetic, and food sectors due to its unique cooling effect and analgesic and anti-inflammatory properties.

Recent advances in plant physiology and molecular biology have unveiled the complex network of pathways involved in the biosynthesis of secondary metabolites like menthol. SLs and SA are key regulators of these pathways. SLs, derived from carotenoids, influence plant architecture and stress responses, while SA, a phenolic compound, is crucial for plant defense [18,19,20,21].

This research investigates the combined effects of SLs and SA on menthol biosynthesis enhancement and the fortification of antioxidant defenses in *Mentha piperita* L. By conducting extensive molecular and biochemical analyses, we aim to identify potential upregulation of genes critical to the menthol biosynthetic pathway and assess the impact on antioxidant enzyme activity. The insights gained from this study could inform strategies to optimize the production of valuable secondary metabolites and improve plant resilience to various stresses.

The enzymatic antioxidants catalase (CAT) and ascorbate peroxidase (APX), along with non-enzymatic antioxidants such as carotenoids and proline, were meticulously quantified to determine the impact of SA and GR24. The study also evaluated the effects on lipid peroxidation and hydrogen peroxide levels, which indicate oxidative stress within plant cells Applying SA and GR24 significantly increased menthol production while decreasing menthofuran and pulegone, compounds associated with lower-quality peppermint oil. The activities of CAT and APX were also elevated, suggesting an upregulation of the plant’s defense mechanisms against oxidative damage.

To test this hypothesis, the study investigated:

- Gene expression in the menthol biosynthetic pathway;
- Essential oil quality;
- Changes in growth and photosynthesis;
- Essential oil yield and quality;
- Antioxidant defense mechanisms.

By examining these factors, this research aims to corroborate the hypothesis that SLs and SA can enhance menthol biosynthesis and bolster the plant’s antioxidative defenses.

## 2. Materials and Methods

### 2.1. Plant Material and Experimental Design

This study aimed to examine the effects of salicylic acid (SA) and strigolactone (SL) on peppermint (*Mentha piperita* L.) plants. Peppermint plants were cultivated in loam soil, previously reported as the optimal soil type for this species by Shukla et al. (1998) [22]. The soil samples were subjected to chemical analysis before the experiment (Table 1). A completely randomized design (CRD) with three replicates was used. The plants were exposed to two levels of SA (0 and 0.1 mM) and GR24 (0 and 10 μM), a synthetic SL analog, applied as foliar sprays at the flowering stage. The plants reached the flowering stage after 60 days of growth, and the hormone treatments were administered. The first spraying occurred 60 days after planting, before the onset of flowering, and the second spraying occurred 24 h before sampling. This timing was selected based on the accumulation of menthol in the flowering stage. A control group was also maintained without any hormone treatment. Uniform peppermint rhizomes were sourced from the Medicinal Plants Research Centre, Institute of Medicinal Plants (ACECR), and planted in pots. The pots were placed in a growth chamber at the Agriculture and Natural Resources of the University of Tehran, Karaj, under a 16/8 h light/dark cycle at 25 ± 1 °C. The above-ground parts of the peppermint were harvested 24 h after the final spraying, and the roots were carefully separated from the soil for further analysis.

### 2.2. Growth Traits

Shoots and roots were weighed at harvest using a digital scale to determine growth traits. In addition, to obtain the dry weight of the samples, they were placed in an oven at 70 °C for 48 h before being weighed.

### 2.3. Carotenoid and Chlorophyll Contents

The extraction of carotenoids and chlorophylls followed Arnon [23]. About 0.1 g of fresh plant leaves was ground with a mortar and pestle in the presence of 1 mL of 80% acetone that contained 200 mg of Na_2_CO_3_. The extract was transferred to a centrifuge tube and centrifuged at 5000 rpm for 5 min. The absorbance of the supernatant was measured at 480, 510, 645, and 663 nm using a spectrophotometer. The concentrations of chlorophyll a, chlorophyll b, total chlorophyll, and carotenoids were calculated with the following formulas:

Chlorophyll a (mg/100 g) = (12.7 × A663 − 2.69 × A645) × V/W

Chlorophyll b (mg/100 g) = (22.9 × A645 − 4.68 × A663) × V/W

Total chlorophyll (mg/100 g) = (20.2 × A645 + 8.02 × A663) × V/W

Carotenoids (mg/100 g) = (1000 × A480 − 3.27 × Chl a − 104 × Chl b) × V/2500 × W

where A is the absorbance at the corresponding wavelength, V is the volume of the extract in mL, and W is the weight of the fresh leaves in g.

### 2.4. Determination of Lipid Peroxidation and Proline Accumulation

Lipid peroxidation was measured by malondialdehyde content (MDA) using the method of Hodges et al. [24] with some modifications. About 0.2 g of crushed leaves were homogenized with 5% trichloroacetic acid (TCA), and the homogenate was centrifuged at 12,000× *g* for 10 min. The supernatant was mixed with 0.5% thiobarbituric acid in 20% TCA, and the mixture was heated in a boiling water bath for 30 min. The mixture was cooled in an ice bath for 5 min and centrifuged again at 12,000× *g* for 10 min. The absorbance of the supernatant was measured at 532 and 600 nm. The MDA content (µmol. mg^−1^ FW) was calculated using an extinction coefficient of 155 mM^−1^cm^−1^. Proline content was measured following Bates et al. [25]. Fresh leaf samples (0.5 g) were homogenized in 10 mL of 3% aqueous sulfosalicylic acid and filtered. Two mL of the filtrate were mixed with 2 mL of acid ninhydrin and 2 mL of glacial acetic acid in a test tube and incubated in a boiling water bath for 1 h. The reaction was terminated by placing the tubes in an ice bath. The absorbance of the reaction mixture was read at 520 nm using a spectrophotometer. A standard curve of L-proline was prepared using the same procedure, and the proline content (nmol g^−1^ FW) was calculated using the regression line equation.

### 2.5. Determination of Hydrogen Peroxide (H_2_O_2_) Accumulation and Total Soluble Phenolic Compounds

The hydrogen peroxide content in the plant samples was determined by the method of Velikova et al. [26]. The dried shoots (0.5 g) were homogenized with 5 mL of cold acetone and then centrifuged at 12,000 rpm for 15 min at 4 °C. The supernatant (0.5 mL) was mixed with 0.5 mL of 10 mM potassium phosphate buffer (pH 7) and 1 mL of 1 M potassium iodide in a test tube and incubated in the dark at room temperature for 10 min. The absorbance of the mixture was measured at 390 nm using a spectrophotometer (UV-1800, Shimadzu, Kyoto, Japan). The hydrogen peroxide content was calculated from a standard curve prepared with H_2_O_2_ and expressed as μmol H_2_O_2_ per g dry weight. The Folin–Ciocalteu reagent estimated the plant extracts’ total phenolic content according to Blainski et al.s’ protocol [27]. In a test tube, 0.5 mL of plant extract was mixed with 2.5 mL of distilled water and 0.5 mL of Folin–Ciocalteu reagent. After 5 min, 1 mL of 20% (*w*/*v*) sodium carbonate solution was added, and the mixture was incubated at room temperature for 30 min. The absorbance of the mixture at 765 nm was recorded using a spectrophotometer. A standard curve was prepared using gallic acid as a reference, and the results were expressed as mg gallic acid equivalent (GAE) g^−1^ dry weight.

### 2.6. Determination of the Activity of Phenylalanine Ammonia Lyase (PAL)

A spectrophotometric method based on the formation of trans-cinnamic acid from L-phenylalanine can be used to measure PAL activity. The principle of this method is that trans-cinnamic acid absorbs light at 290 nm, and the increase in absorbance over time reflects the rate of the PAL-catalyzed reaction. The technique requires a reaction mixture containing L-phenylalanine as the substrate, borate buffer to maintain the pH at 8.8, and plant extract as the enzyme source. The reaction is initiated by adding the plant extract to the mixture and incubating it at 30 °C for 30 min. The reaction is stopped by adding trichloroacetic acid, precipitating the protein in the plant extract. The supernatant is then transferred to a cuvette, and the absorbance is measured at 290 nm using a spectrophotometer. The amount of trans-cinnamic acid produced can be calculated from a standard curve prepared with known concentrations of trans-cinnamic acid. The enzyme activity can be expressed as nanomoles of trans-cinnamic acid produced per minute per milligram of protein (Beaudoin-Eagan and Thorpe, 1985) [28].

### 2.7. Measurement of Catalase and Ascorbate Peroxidase Activities

To assess the oxidative stress and antioxidant defense in plants, the activities of two key enzymes in the ascorbate–glutathione cycle were measured: catalase (CAT, EC 1.11.1.6) and ascorbate peroxidase (APX, EC 1.11.1.11). CAT protects cells from oxidative damage by decomposing hydrogen peroxide (H_2_O_2_) into water and oxygen, while APX reduces H_2_O_2_ to water using ascorbate as an electron donor. Both enzymes are sensitive indicators of the effects of salicylic acid and synthetic strigolactone in peppermint.

CAT activity was determined by the decrease in absorbance at 240 nm due to the consumption of H_2_O_2_ in a reaction mixture containing 50 mM phosphate buffer (pH 7.0), plant extract as the enzyme source, and H_2_O_2_ as the substrate. The reaction was initiated by adding H_2_O_2_ and incubated at 25 °C for 3 min. The residual H_2_O_2_ concentration was calculated from a standard curve prepared with known concentrations of H_2_O_2_. CAT activity was expressed as μmol of H_2_O_2_ decomposed per min per mg of protein [29].

APX activity was determined by the decrease in absorbance at 290 nm due to ascorbate oxidation in a reaction mixture containing 50 mM phosphate buffer (pH 7.0), ascorbate, and H_2_O_2_ in a final volume of 3 mL. The reaction was started by adding 10 μL of enzyme extract and incubated at 25 °C. The amount of ascorbate oxidized was calculated from the extinction coefficient of 2.8 mM^−1^ cm^−1^ at 290 nm. One unit of APX activity was defined as the amount of enzyme that oxidized 1 μmol of ascorbate per min at 25 °C. APX activity was expressed as U per mg of protein [30].

### 2.8. DPPH Radical Scavenging Activity

Brand-Williams et al. [31] used the method to measure the DPPH radical scavenging activity of the plant extracts. The dried shoots (0.5 g) were sonicated in 10 mL of methanol for 15 min and then filtered. The filtrate (0.1 mL) was added to a test tube containing 3.9 mL of methanol and 0.5 mL of 0.1 mM DPPH solution. The mixture was incubated in the dark at room temperature for 30 min. The absorbance of the mixture at 517 nm was measured using a spectrophotometer (UV-1800, Shimadzu, Kyoto, Japan). The percentage of DPPH reduction was calculated as DPPH radical scavenging activity (%) = [(A0 − A1)/A0] × 100, where A0 is the absorbance of the control (methanol + DPPH solution), and A1 is the absorbance of the sample (extract + methanol + DPPH solution).

### 2.9. Extraction and Identification of Essential Oil Compounds

The dried shoots (0.5 g) were prepared for essential oil extraction by drying in a shaded area at room temperature to preserve the oil quality [32]. The shoots were then subjected to hydrodistillation for four hours using a Clevenger apparatus, which separates and collects the oil from the water by condensation [33]. The volume and yield of the extracted oil were measured.

The chemical composition of the essential oil was determined using gas chromatography–mass spectrometry (GC-MS). An Agilent 6890 series GC system equipped with an Agilent 5973 mass selective detector and an HP-5MS capillary column (30 m × 0.25 mm × 0.25 μm) was used for the analysis. The GC conditions were as follows: The injector temperature was set at 250 °C, and the oven temperature was initially held at 60 °C for 10 min, then increased to 240 °C at a rate of 3 °C per minute. Helium was used as the carrier gas at a 1 mL/min flow rate, with a split ratio of 1:50. A 1 μL aliquot of the essential oil was injected into the GC-MS system. The mass spectrometer was operated in electron ionization mode, with an ionization energy of 70 eV, and the mass scan range was set from 40 to 550 *m*/*z*.

The essential oil compounds were identified by comparing their retention indices and mass spectra with those of authentic standards and reference data from the NIST (National Institute of Standards and Technology) library. The retention index indicates the retention time of a compound in the column relative to a series of standard hydrocarbons [34]. The relative percentage of each compound was calculated based on the peak area in the chromatogram.

### 2.10. Gene Expression Analysis

TheGeneAll^®^ Ribospin™ kit (BioFrontier, Seoul, Republic of Korea) was used to extract the total RNA of peppermint samples. The Revert Aid™ First Strand cDNA Synthesis Kit (Fermentas, Waltham, MA, USA) synthesized complementary DNA (cDNA) from the extracted RNA. Online primer Quest software (version 2.2.3) designed primers for the selected genes that encode pulegone reductase (*pr*), menthofuran synthase (*MFS*), and *MDH* based on cDNA sequences (Table 2). The actin gene served as an internal standard for transcript analysis. A Step One Plus machine (ABI, New York, NY, USA) and a HIFI SYBR Green kit performed RT-PCR on the cDNA. The RT-PCR program consisted of two steps: The first step was 180 s at 95 °C, and the second step was 10 s at 95 °C, 10 s at 59 °C, and 30 s at 72 °C for 35 cycles.

#### Data Analyses

The data were analyzed using RStudio software (version 4.3.2). The Duncan test was performed at a 5% significance level to compare the means. Mathematical models from Pfaffl et al. [35] were applied to the real-time PCR data.

Cluster analysis was conducted to group the samples based on physiological and biochemical responses to different phytohormone treatments. Hierarchical clustering was performed using Ward’s method, and Euclidean distance was implemented in R software (version 4.3.2) to measure similarity. The resulting dendrogram was used to visualize the clustering patterns among the samples.

Correlation analysis was carried out to examine the relationships between physiological and biochemical adaptations in plants subjected to varying phytohormone treatments. Spearman correlation coefficients were calculated to determine the strength and direction of these associations. The correlation matrix was visualized using a heatmap to highlight significant correlations.

## 3. Results

### 3.1. Effects of Salicylic Acid and Strigolactone on Growth Parameters of Peppermint Plants

**Figure 1:**
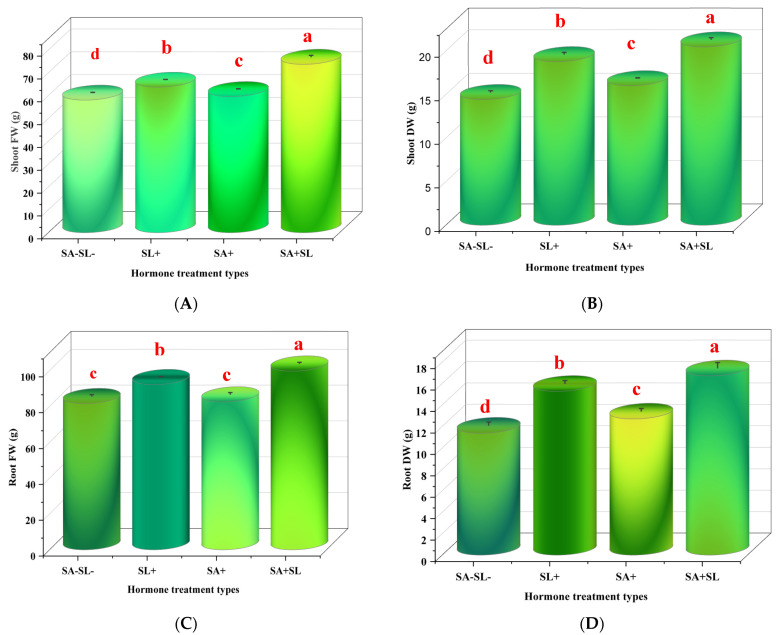
Effect of phytohormone application on growth traits of peppermint plants. Peppermint plants were grown in loam soil for 60 days. At the flowering stage, the plants were sprayed with two levels of SA (0 and 0.1 mM) and two levels of GR24 (0 and 10 μM) twice, 60 days after planting and 24 h before sampling. (**A**) Shoot fresh weight (FW). (**B**) Shoot dry weight (DW). (**C**) Root FW. (**D**) Root DW. Error bars indicate standard error (SE). Different letters within a column indicate a significant difference (Duncan, *p* < 0.05, n = 3).

The application of SA and SL, individually and in combination, increased the fresh and dry weights of peppermint shoots and roots. The combination treatment (SA + SL) exhibited the most significant enhancement in growth parameters, with a statistically significant increase (*p* < 0.05) compared to the control and other treatments. Among the individual treatments, SL alone had a more pronounced effect on growth than SA alone (Figure 1).

### 3.2. Improvement in Photosynthetic Pigment Content in Peppermint by Foliar Application of Salicylic Acid and Strigolactone

**Figure 2:**
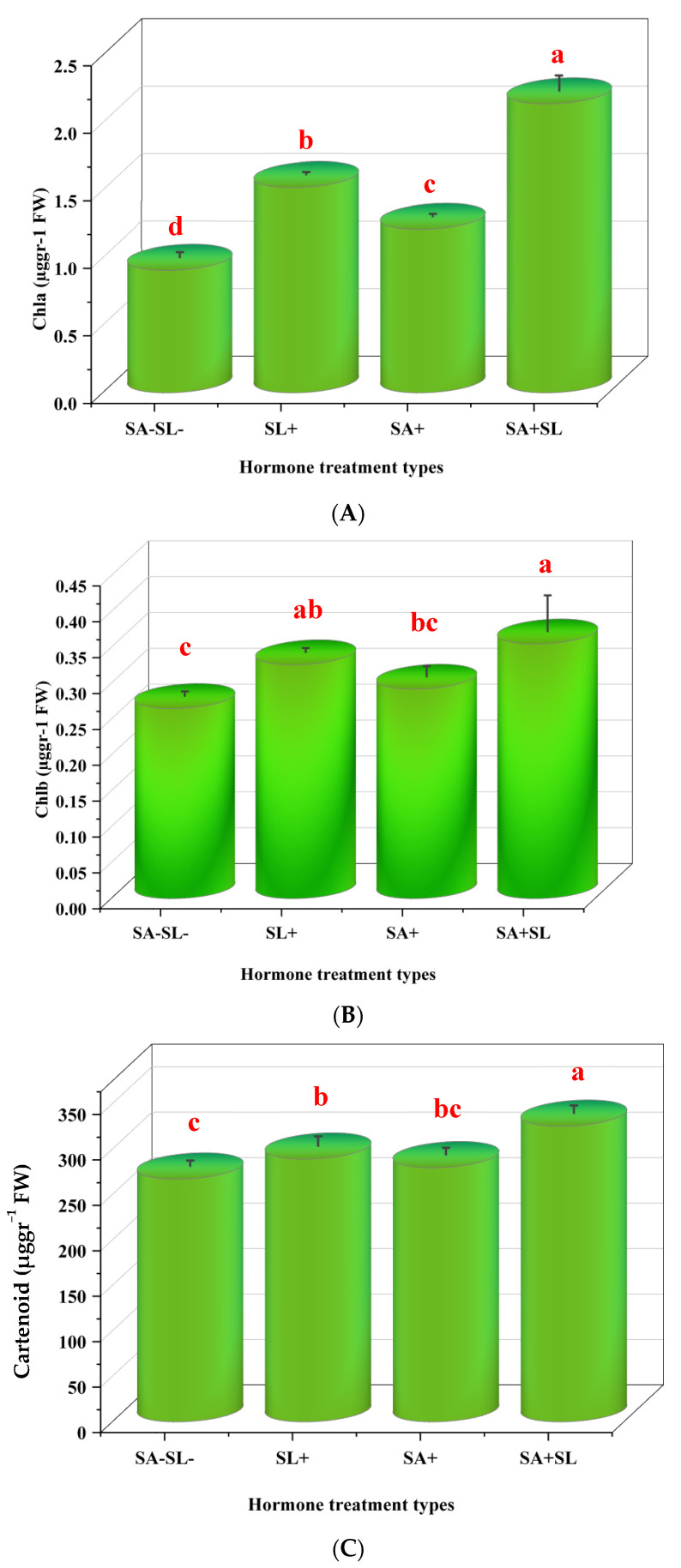
Effect of phytohormone application on chlorophyll content of peppermint plants. (**A**) Chlorophyll a content. (**B**) Chlorophyll b content. (**C**) Carotenoid content. Error bars indicate standard error (SE). Different letters within a column indicate a significant difference (Duncan, *p* < 0.05, n = 3). For detailed growth conditions, refer to Figure 1.

The combined application of SA and GR24 resulted in a significant increase in the content of chlorophyll a, chlorophyll b, and carotenoids. The combination treatments showed a more pronounced effect compared to the control. Notably, GR24 alone had a significantly more significant impact on pigment content than SA alone, with a statistically significant difference at the 5% level (*p* < 0.05) (Figure 2).

### 3.3. Effects of SA and GR24 on H_2_O_2_, MDA, Proline, and Total Soluble Phenolic Levels

The experimental results indicated that the control group, which was not treated with salicylic acid (SA) or strigolactone (SL), exhibited elevated levels of hydrogen peroxide (H_2_O_2_) and malondialdehyde (MDA). Subsequent treatments revealed that the application of SA alone resulted in higher H_2_O_2_ and MDA levels than the SL-alone treatment. However, the combined treatment of SA and SL demonstrated a synergistic effect, reducing H_2_O_2_ and MDA levels to below those observed in the individual SA or SL treatments.

Conversely, the total phenolic content and proline levels were augmented in the presence of SL as a standalone treatment and in combination with SA. The combined application of SA and SL was particularly effective, yielding the highest increase in these compounds. The SL-alone treatment followed closely, surpassing the effect of SA alone. The control group showed the lowest concentrations of total phenolics and proline, underscoring the impact of the treatments.

**Figure 3:**
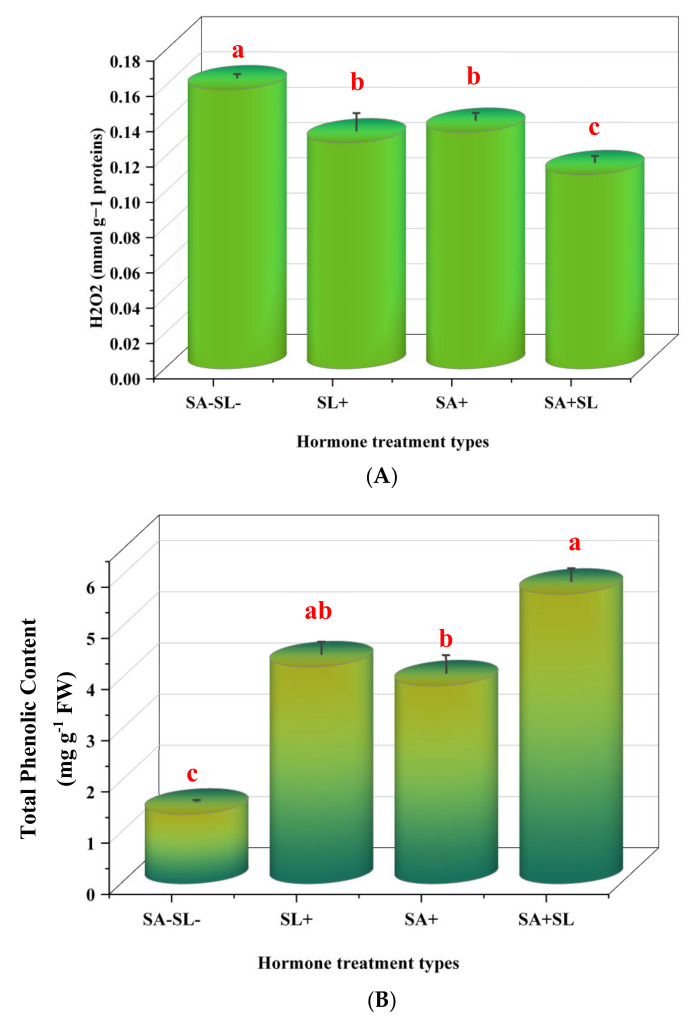

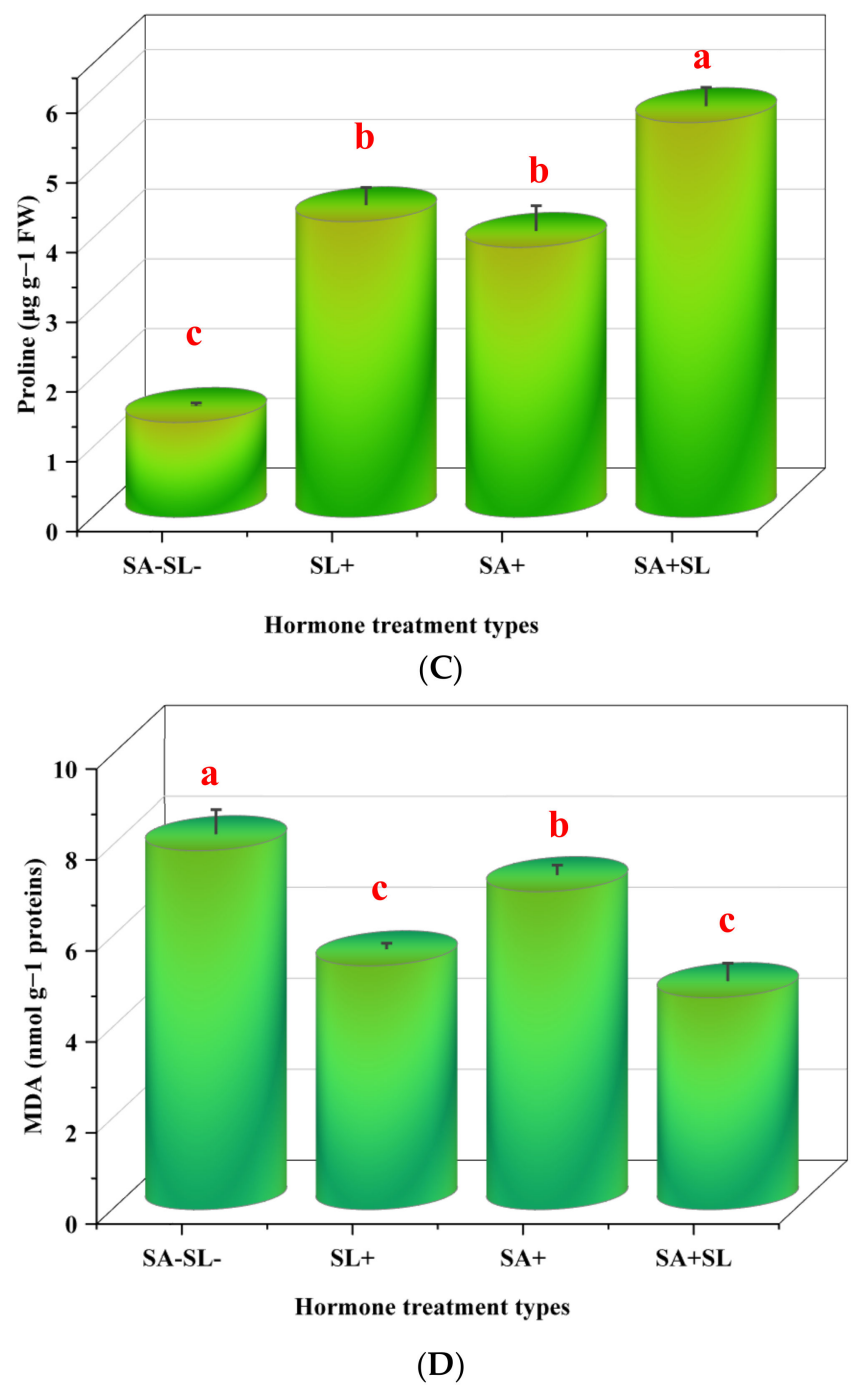
Effect of phytohormone application on peppermint plants’ proline, H_2_O_2_, total phenolic, and MDA content. (**A**) H_2_O_2_ content. (**B**) Proline content. (**C**) Total phenolic content. (**D**) MDA content. Error bars indicate standard error (SE). Different letters within a column indicate a significant difference (Duncan, *p* < 0.05, n = 3). For detailed growth conditions, refer to Figure 1.

These data suggest that the co-application of SA and SL could be a potent strategy for enhancing plant antioxidant defense mechanisms, as evidenced by lower stress markers (H_2_O_2_ and MDA) and higher concentrations of protective compounds, total phenolics, and proline (Figure 3).

### 3.4. Effects of Strigolactone and Salicylic Acid on Antioxidant Enzymes, Phenolic Compounds, and Radical Scavenging Activity in Peppermint

**Figure 4:**
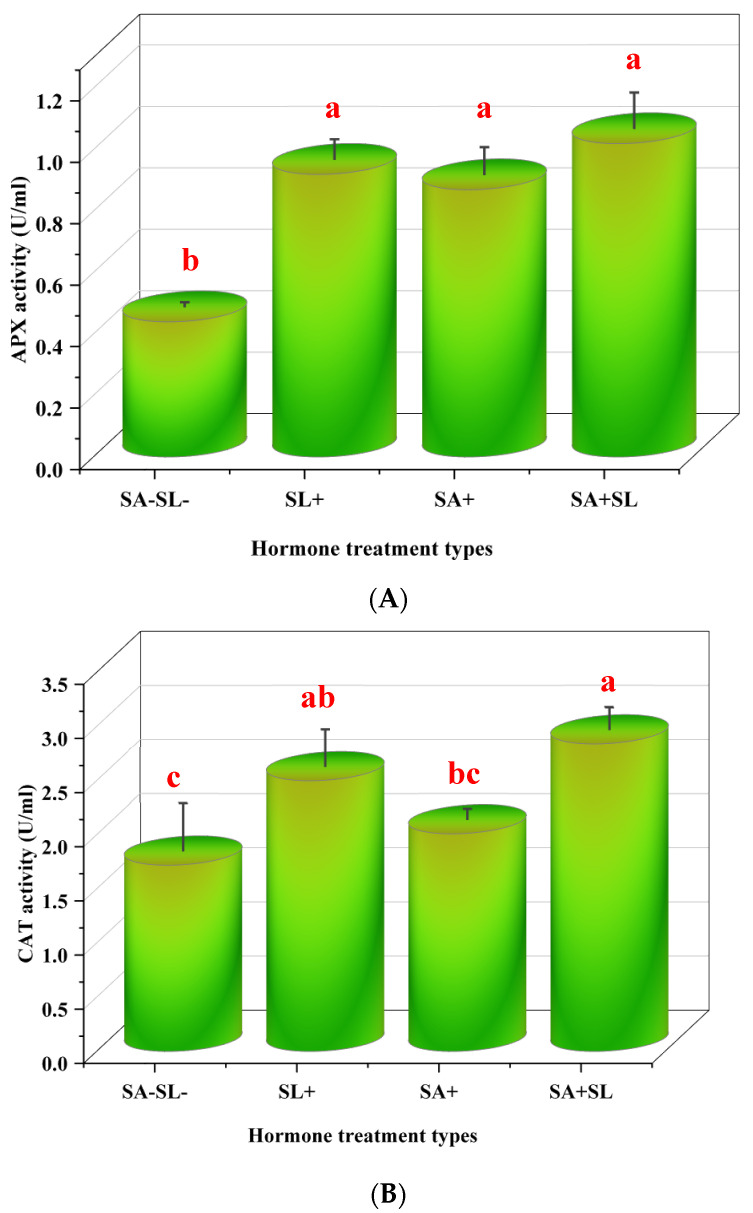

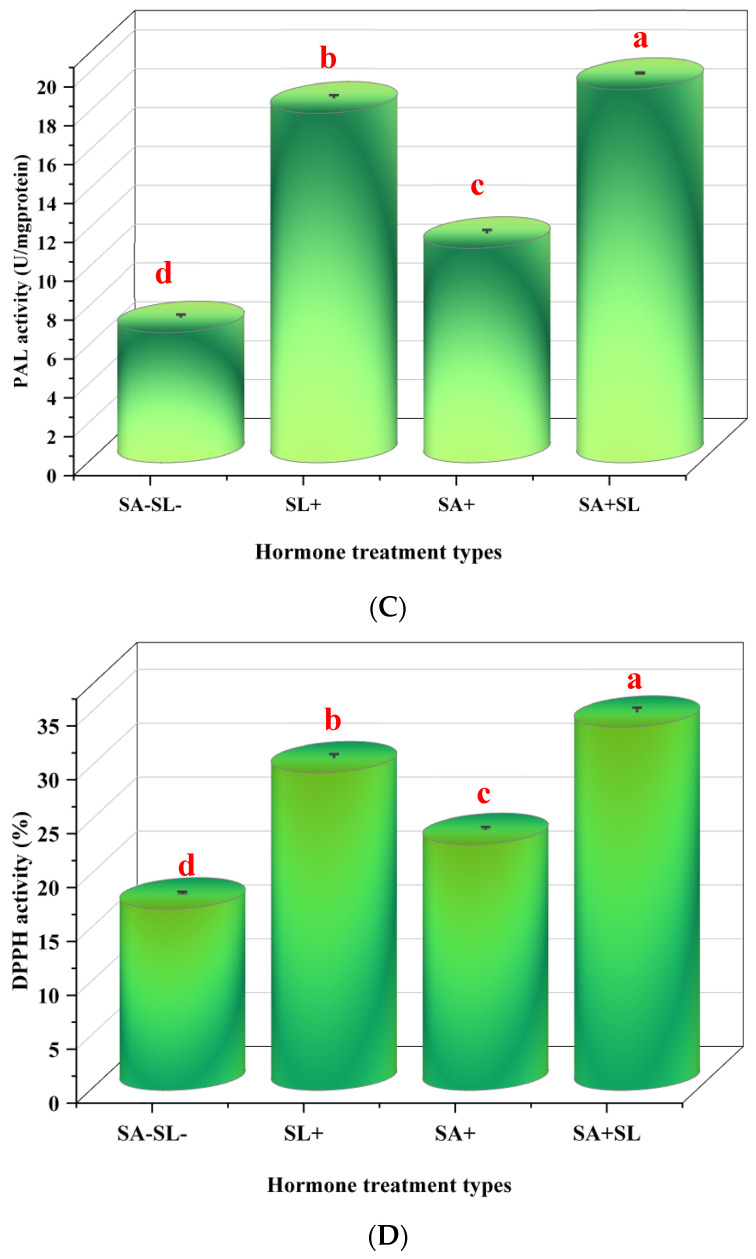
Effects of GR24 and SA on peppermint plants’ antioxidant enzymes and phenolic compounds. (**A**) Ascorbate peroxidase (APX) activity. (**B**) Catalase (CAT) activity. (**C**) Phenylalanine ammonia lyase (PAL) activity. (**D**) DPPH radical scavenging activity. The values are means ± SD of three replicates. Different letters within a column indicate a significant difference (Duncan, *p* < 0.05, n = 3). For detailed growth conditions, refer to Figure 1.

This study investigated the influence of SL and SA on the activity of antioxidant enzymes and the content of phenolic compounds in *Mentha piperita* L. The enzymatic activities of ascorbate peroxidase (APX) and catalase (CAT), along with the phenylalanine ammonia lyase (PAL) activity and 2,2-diphenyl-1-picrylhydrazyl (DPPH) radical scavenging activity, were measured. The results indicated that the combined application of SA and SL led to a synergistic increase in APX, CAT, and PAL activities and significantly enhanced DPPH radical scavenging capacity. Notably, the application of SL alone resulted in higher enzyme activities and DPPH scavenging, APX, CAT, and PAL activities compared to the application of SA alone. Control treatments, which did not receive SA or SL, exhibited the lowest levels of enzyme activities and DPPH radical scavenging capacity. This suggests that both SA and SL play a significant role in bolstering the antioxidant defense system in peppermint, with SL having a more pronounced effect when applied independently (Figure 4).

### 3.5. Effects of Phytohormones on the Chemical Composition of Peppermint Essential Oi

**Figure 5:**
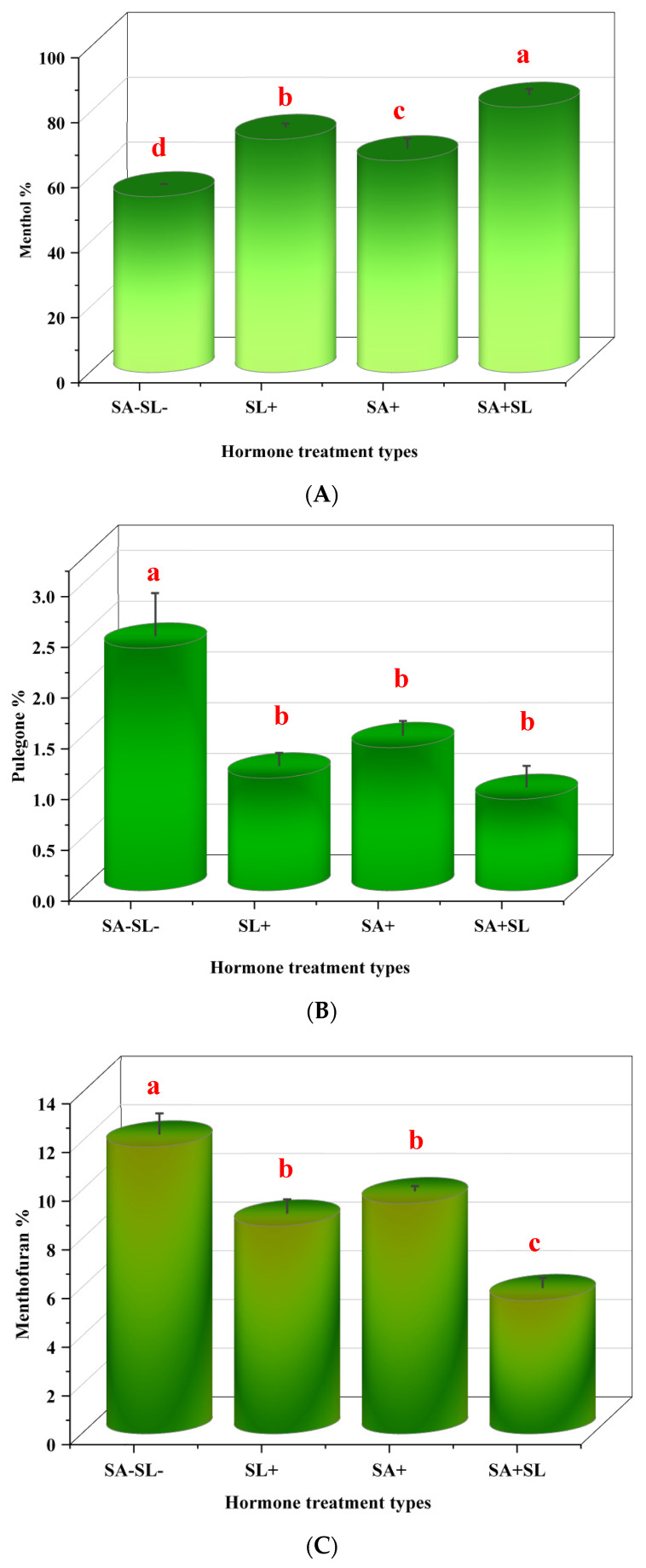
Effects of GR24 and SA on menthol, pulegone, and menthofuran content in peppermint plants. (**A**) Menthol content. (**B**) Pulegone content. (**C**) Menthofuran content. Different letters within each column indicate significant differences (Duncan’s multiple range test, *p* < 0.05, n = 3). For detailed growth conditions, refer to Figure 1.

The application of SA and synthetic GR24, individually or in combination, significantly influenced the menthol content in *Mentha piperita* L. The combined treatment of SA and GR24 resulted in the highest menthol accumulation, surpassing the effects observed with GR24 or SA applied separately. GR24 alone was the second most effective treatment in enhancing menthol levels, followed by SA alone, with the control group exhibiting the lowest menthol content. Conversely, the levels of pulegone and menthofuran were elevated in the control plants, which did not receive any phytohormone treatment. The SA application led to a higher concentration of these compounds among the treated groups than the GR24 treatment. These data suggest that the synergistic interaction between SA and GR24 can be harnessed to modulate the essential oil profile of peppermint, particularly by augmenting menthol synthesis while controlling the levels of pulegone and menthofuran (Figure 5).

### 3.6. Correlation Analysis

**Figure 6:**
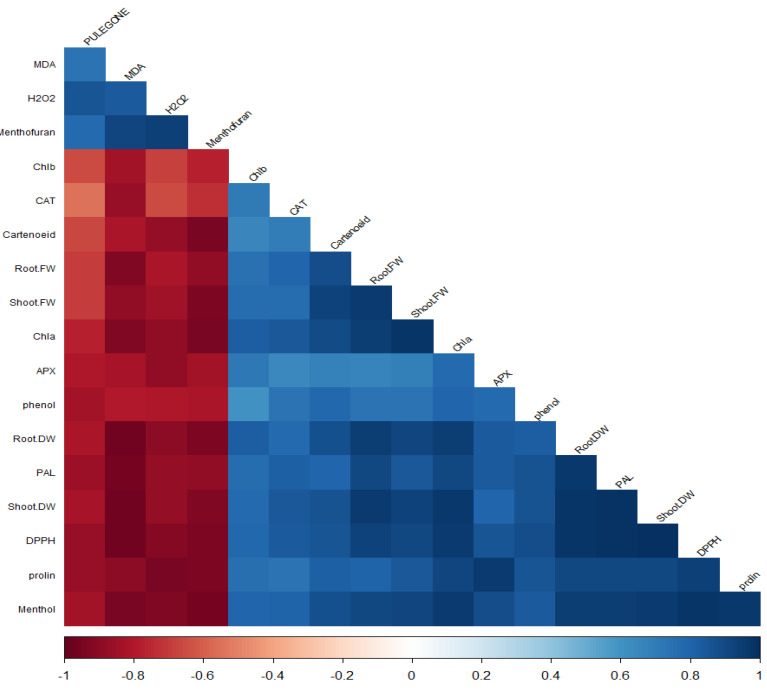
Correlation analysis of physiological and biochemical adaptations in plants subjected to varying phytohormone treatments.

The correlation analysis revealed significant associations among various phytochemicals and plant physiological parameters (Figure 6). A robust correlation between menthol and chlorophyll a (Chl a) indicated a possible link between menthol synthesis and photosynthetic capacity. Similarly, strong positive correlations were identified between shoot fresh weight (FW) and root FW, as well as between root dry weight (DW) and phenylalanine ammonia lyase (PAL) activity, suggesting a coordinated growth and defense response. Additionally, shoot DW was highly correlated with DPPH radical scavenging activity and proline content, implying a relationship between biomass accumulation and antioxidant defense mechanisms. Furthermore, hydrogen peroxide (H_2_O_2_) levels showed significant correlations with pulegone and malondialdehyde (MDA) content, while MDA also correlated with pulegone, indicating oxidative stress responses. Menthofuran demonstrated correlations with MDA and H_2_O_2_, suggesting its involvement in the plant’s response to oxidative stress. Menthofuran was associated with MDA, H_2_O_2_, and pulegone, reinforcing the potential interplay between these metabolites under stress conditions.

Proline, a well-known osmoprotectant, was highly correlated with ascorbate peroxidase (APX) activity, Chla, root DW, PAL, and shoot DW, highlighting its multifaceted role in plant stress tolerance. The strongest associations for proline were with Chla, APX, root DW, PAL, and shoot DW, underscoring its significance in photosynthetic function and stress adaptation. Interactions between root FW and carotenoids and correlations between shoot FW and carotenoids were noted, suggesting a link between biomass and pigmentation. Chlorophyll also correlates with carotenoids, root FW, and shoot FW, indicating a concerted action between photosynthetic pigments and growth parameters.

High correlations were found between root DW and root FW, carotenoids, shoot FW, and Chl a, reflecting the integrated nature of plant growth and pigment content. PAL activity showed significant correlations with root FW, Chl a, and root DW, pointing to its role in phenylpropanoid metabolism and its association with growth and photosynthesis. Lastly, shoot DW was highly correlated with root FW, shoot FW, Chla, root DW, and PAL, suggesting a complex interaction between biomass production, photosynthetic efficiency, and secondary metabolism. This comprehensive analysis underscores the intricate network of relationships that govern plant metabolism and stress responses, providing valuable insights into plants’ physiological and biochemical adaptations under varying conditions.

### 3.7. Cluster Analysis

The cluster analysis revealed distinct groupings among the variables studied. Initially, pulegone and MDA formed the first cluster, which subsequently grouped with H_2_O_2_ and menthofuran to create a unified cluster. APX and proline were clustered, while phenol was isolated in its cluster. Photosynthetic pigments such as chlorophyll a (Chl a), carotenoids, and shoot fresh weight (FW) were grouped in a single cluster. This was followed by a cluster combining shoot FW and dry weight (DW). A comprehensive cluster encompassed shoot DW, catalase (CAT), chlorophyll b (Chl b), DPPH, menthol, and phenylalanine ammonia lyase (PAL) alongside root FW and DW.

**Figure 7:**
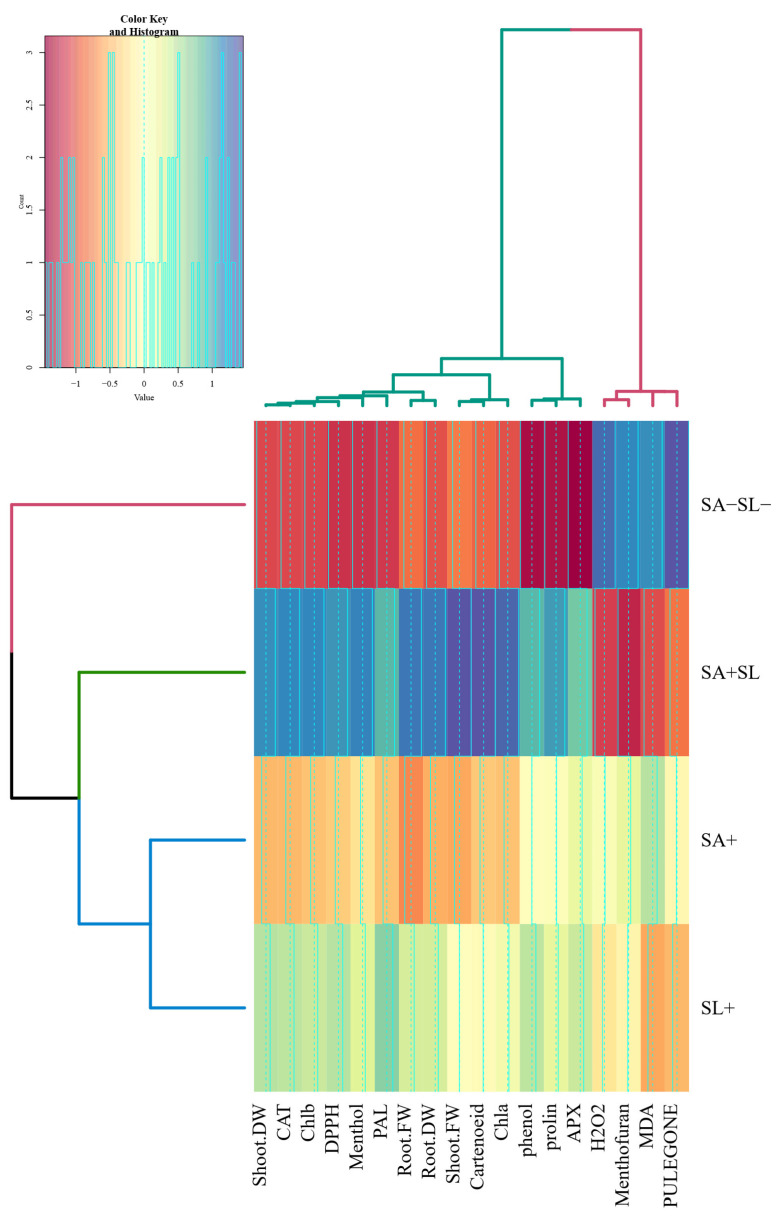
Cluster analysis of physiological and biochemical responses in peppermint to different phytohormone treatments.

Furthermore, treatments involving salicylic acid (SA) and sodium lignosulfonate (SL) were clustered together, which then joined the cluster comprising SA + SL treatment, and ultimately, these categories were linked to the control group. Notably, traits such as Chla, carotenoids, shoot FW, root FW, menthol, DPPH, Chlb, CAT, and shoot DW exhibited higher values in the SA + SL treatment. Conversely, higher levels of pulegone, MDA, menthofuran, and H_2_O_2_ were observed in the control group (Figure 7).

### 3.8. Differential Expression of Genes Related to Menthol Biosynthesis

The study employed quantitative real-time PCR (qRT-PCR) to evaluate the influence of phytohormone treatments on the expression of key genes central to menthol biosynthesis: (*MDH*), pulegone reductase (*PR*), and menthofuran synthase (*MFS*). MD is instrumental in converting geranyl diphosphate into menthofuran, a precursor in menthol synthesis. *MDH* is crucial in reducing menthofuran to menthol, while *PR* oversees the conversion of menthol to pulegone.

Differential gene expression was observed under various treatment conditions. The combined application of GR24 and SA markedly upregulated the expression of *PR* and *MDH*. Conversely, the absence of SA and SL was associated with reduced gene expression. Control samples lacking SA and SL demonstrated increased MFS expression, with SA treatment alone eliciting a more significant expression than SL. However, the simultaneous treatment with SA and SL led to a decrease in expression levels.

These observations suggest that GR24 and SA treatments enhance the downstream steps in the monoterpene biosynthesis pathway, leading to increased menthol production. The synergistic effect of GR24 and SA was particularly evident in the upregulation of *MDH* and *PR*, indicating a potential cooperative interaction between these phytohormones. The levels of pulegone and menthofuran, products of PR and MFS, respectively, agreed with their gene expression patterns, confirming the modulatory effect of GR24, SA, and their combined treatment on the biosynthesis of these metabolites.

Furthermore, the suppression of *MFS* expression and the reduction in menthofuran accumulation was found to increase *PR* expression and decrease pulegone levels. This aligns with previous findings that phytohormones can modulate terpenoid biosynthesis by modifying gene expression and accumulating secondary metabolites [36,37]. Therefore, phytohormones may enhance secondary metabolite production in stressed plants.

**Figure 8:**
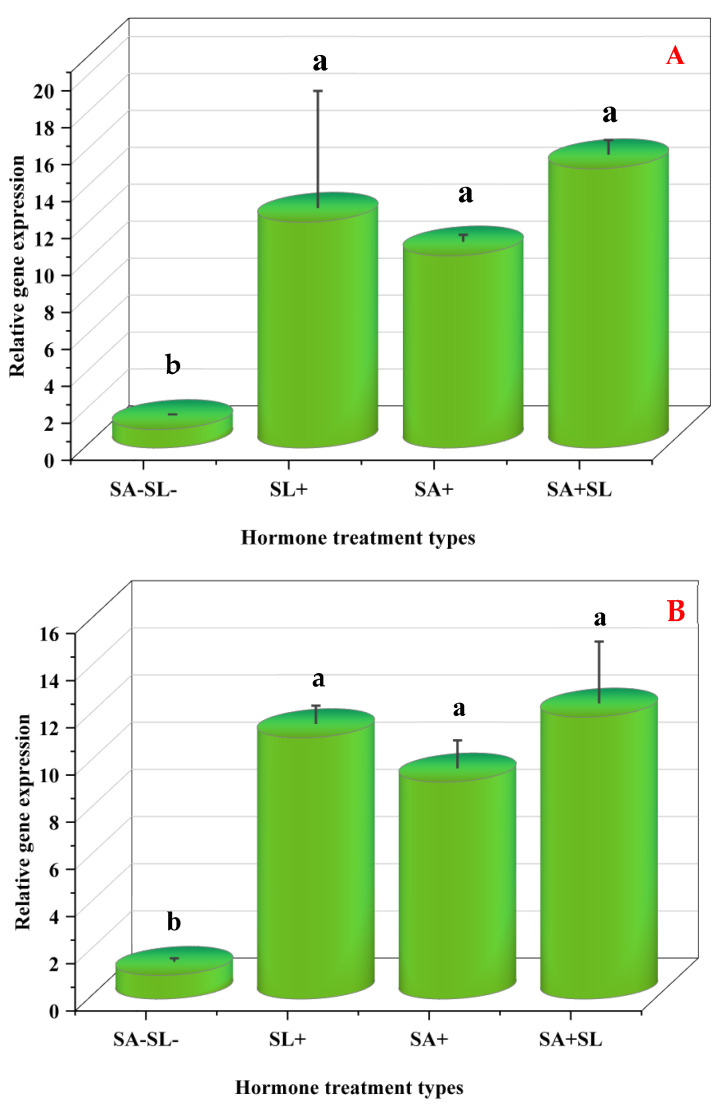

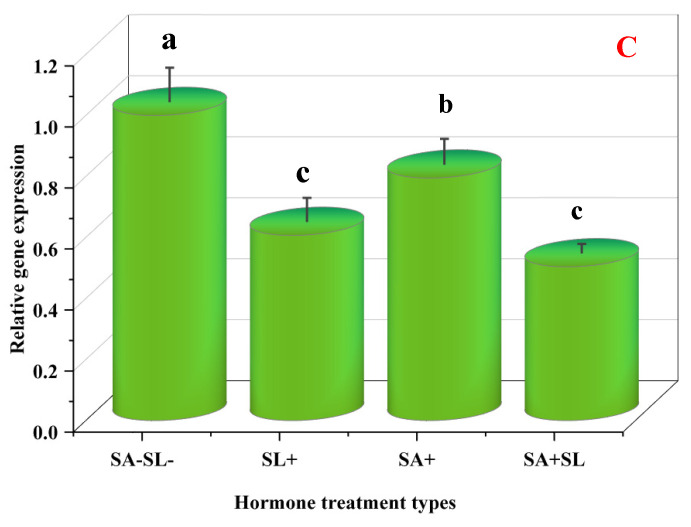
Changes in the expression levels of three genes involved in menthol biosynthesis of peppermint plants. (**A**) Menthol dehydrogenase (*MDH*). (**B**) Pulegone reductase (*PR*). (**C**) Menthofuran synthase (*MFS*). Different letters within a column indicate a significant difference (Duncan, *p* < 0.05, n = 3). For detailed growth conditions, refer to Figure 1.

In summary, the transcriptional regulation of *MDH*, *PR*, and *MFS* by GR24 and SA and their interplay within the SL-SA pathways presents a promising avenue for metabolic engineering or breeding approaches aimed at improving menthol production or enhancing metal tolerance in medicinal plants through the increased synthesis of secondary metabolites (Figure 8).

## 4. Discussion

The results of this study indicate that the foliar application of SA and SL significantly enhances the growth parameters of peppermint plants. Specifically, the combination of SA and SL resulted in the highest increase in both fresh and dry weights of shoots and roots. These findings suggest that SA and SL can synergistically promote plant growth. Previous studies have demonstrated the individual roles of SA and SL in plant growth and development. For instance, Ref. [38] reported that SA application improved the growth and yield of various crops, including wheat and soybean. Similarly, SLs have been identified as essential regulators of plant architecture and stress responses [39,40,41]. According to [42], SLs influence root development, shoot branching, and the plant’s response to nutrient availability.

The observed increase in growth parameters in this study aligns with the findings of these previous studies. The combined application of SA and SL appears to amplify their individual effects, leading to a more pronounced growth enhancement. This synergistic effect may be attributed to the complementary roles of SA and SL in regulating plant physiological processes. SA is known to modulate the plant’s hormonal balance and activate defense mechanisms [43,44], while SLs primarily influence plant architecture and resource allocation [45].

Moreover, the results of this study are consistent with the findings of [46,47], who reported that the combined application of SA and SLs improved the growth and yield of tomato plants similarly, demonstrated that the co-application of SA and SLs enhanced the growth and stress tolerance of rice plants. These studies support the hypothesis that SA and SLs can improve plant growth and productivity.

In addition, the foliar application of SA and GR24, a synthetic analog of strigolactone (SL), has significantly impacted the enhancement of photosynthetic pigments in peppermint (*Mentha piperita*). This study’s findings align with the known roles of SA and SL in plant stress responses and development. SA is recognized for its role in local and systemic acquired resistance against pathogens and in acclimation to abiotic stressors, which may include modulation of photosynthetic processes [48,49]. Similarly, SLs are involved in plant growth and stress mitigation [50].

The observed increase in chlorophyll a, chlorophyll b, and carotenoids upon treatment with SA and GR24 suggests a synergistic effect of these hormones on pigment biosynthesis. The combination of SA and GR24 is more effective than the application of SA alone. This is consistent with the notion that SL may have a more pronounced influence on photosynthetic efficiency under stress conditions. This is further supported by the fact that SL alone had a more significant effect than SA alone, indicating that SL might play a more direct role in the biosynthesis or stabilization of photosynthetic pigments.

Comparatively, the application of SA has been shown to have variable effects on photosynthesis, contingent on factors such as plant species, environmental conditions, and concentration. In some cases, SA may act as a stress factor, negatively influencing physiological processes, while in others, it may alleviate stress effects, potentially leading to higher photosynthetic capacity. Peppermint’s positive response to SA in this study could be attributed to the specific concentration used, which did not reach a level that would induce stress in the plants.

Enhancing photosynthetic pigments is crucial for plant productivity, as these pigments are directly involved in capturing light energy for photosynthesis. Increasing pigment content could imply an improved photosynthetic capacity, which may translate to higher biomass production and potentially greater yields of essential oils, which are economically important in peppermint cultivation.

It is also noteworthy to compare these results with other studies where different plant hormones, such as jasmonic acid and brassinosteroid, have been applied to peppermint under various stress conditions, leading to changes in antioxidant activity and essential oil composition [51]. These studies highlight the complex interplay between plant hormones and environmental stressors and their collective impact on plant physiology and secondary metabolite production.

This study elucidated the interplay between SA and SL, particularly GR24, on the oxidative stress markers and antioxidant defense compounds in *Mentha piperita* L. Our findings align with the growing body of literature that underscores the role of phytohormones in modulating plant responses to environmental stresses [52,53,54].

The elevated levels of H_2_O_2_ and MDA in the control group indicated oxidative stress, which is consistent with previous reports. The application of SA alone led to increased H_2_O_2_ and MDA, suggesting that SA at the given concentration might induce oxidative stress rather than alleviate it. This contrasts the role of SA as a signaling molecule in plant defense, as reported by Rivas-San Vicente and Plasencia [55].

Interestingly, the SL-alone treatment reduced the levels of H_2_O_2_ and MDA, which agrees with studies demonstrating the antioxidant potential of SLs5. The synergistic effect observed upon the combined application of SA and SL, leading to even lower levels of H_2_O_2_ and MDA, suggests a complex interaction between these two phytohormones that enhances the plant’s antioxidant defense. This synergism has been previously observed, where the combined application of SA and SL resulted in improved drought tolerance in wheat through the modulation of antioxidant enzymes.

Furthermore, the increase in total soluble phenolics and proline levels upon SL treatment, both alone and in combination with SA, indicates an enhanced synthesis of these protective compounds. Phenolics are known for their role in scavenging reactive oxygen species, while proline acts as an osmoprotectant, stabilizing proteins and membranes under stress conditions [56]. Following the combined treatment, the fact that these compounds showed the highest increase suggests that the interaction between SA and SL could be exploited to bolster the plant’s resilience to stress.

Comparatively, the control group’s lower concentrations of total phenolics and proline underscore the impact of the treatments and the necessity of phytohormonal intervention for improved stress tolerance. The results from this study contribute to the understanding of SA and SL’s roles in plant stress physiology and offer a promising strategy for enhancing antioxidant defenses in *Mentha piperita* L.

The results of this study highlight the significant role of SL and SA in enhancing the antioxidant defense mechanisms in *Mentha piperita* L. The combined application of SA and SL synergistically increased the activities of APX, CAT, and phenylalanine ammonia lyase (PAL) and the DPPH radical scavenging capacity. This suggests a cooperative interaction between these two signaling molecules to bolster the plant’s defense system against oxidative stress.

The observed increase in antioxidant enzyme activity and radical scavenging activity with SL alone, compared to SA alone, is consistent with findings from [57], who reported that the exogenous application of GR24, a synthetic analog of SL, enhanced antioxidant enzyme activity in cucumber seedlings under low-light stress. This supports the notion that SLs may be more dominant in the antioxidant response than previously understood.

Furthermore, the interaction between SL and SA in plant defense responses has been documented by [58], who noted that SLs could induce the production of SA, thereby activating plant defense responses. This crosstalk between SL and SA pathways is crucial for the plant’s ability to adapt to environmental stresses and maintain homeostasis.

The priming effect of SLs on plant defense, mainly through SA-mediated signaling, has been demonstrated in *Arabidopsis thaliana* [59]. The application of GR24 enhanced disease resistance, suggesting that SLs may have a broader role in plant defense beyond their direct antioxidant effects, potentially involving the modulation of SA-mediated pathways.

In light of these findings, SLs are potent modulators of plant defense responses, particularly in enhancing antioxidant capacity and interacting with SA signaling pathways. The current study adds to the growing body of evidence supporting the potential use of SLs to bolster plant resilience against environmental stresses, which could have significant implications for agriculture and crop improvement.

The impact of phytohormones on the chemical composition of peppermint essential oil has been a subject of considerable interest within plant biochemistry. Our study focused on the effects of SA and synthetic GR24 on the primary constituents of peppermint essential oil, revealing a synergistic effect when both phytohormones are applied together, particularly in enhancing menthol content. This finding is consistent with the known roles of SA and GR24 in plant growth and stress responses, where they modulate gene expression and secondary metabolite production.

The elevated menthol levels observed in the SA and GR24 combined treatment could be attributed to the enhanced gene expression in the menthol biosynthesis pathway. This aligns with previous research demonstrating that phytohormones can influence the expression of key biosynthetic genes, thereby altering the plant’s secondary metabolite profile. For instance, studies have shown that jasmonic acid, another phytohormone, can upregulate genes in the menthol pathway, suggesting a complex interplay between different phytohormones in regulating essential oil composition.

The current investigation into the differential expression of genes associated with menthol biosynthesis has revealed a complex interplay between phytohormones and gene regulation. The upregulation of menthol dehydrogenase (MD) and pulegone reductase (PR) in response to GR24 and salicylic acid (SA) treatments underscores the potential of these phytohormones to enhance menthol production through the monoterpene biosynthesis pathway. This synergistic effect is particularly noteworthy, as it suggests a cooperative interaction that could be harnessed in metabolic engineering strategies.

The observed decrease in menthofuran synthase (MFS) expression under specific treatment conditions aligns with the concept that phytohormones can modulate the biosynthesis of terpenoids, vital secondary metabolites in plants. This modulation is crucial for plants, especially under stress conditions, where the production of secondary metabolites can contribute to stress tolerance and survival. Comparatively, our findings resonate with those of [60], who reported that chitosan, gibberellic acid, and methyl jasmonate treatments influenced the expression of genes in the menthol biosynthesis pathway in peppermint.

## 5. Conclusions

The present investigation offers compelling evidence that salicylic acid (SA) and synthetic strigolactone (GR24) act as potent bio-stimulants, significantly enhancing menthol production in *Mentha piperita* L. while simultaneously mitigating the synthesis of undesirable compounds such as menthofuran and pulegone. The upregulation of enzymatic antioxidants catalase (CAT) and ascorbate peroxidase (APX), in conjunction with increased levels of non-enzymatic antioxidants, underscores a fortified antioxidant defense system. The observed reduction in lipid peroxidation and hydrogen peroxide levels further corroborates this. The differential expression of the menthofuran synthase (*MFS*) gene in the control group, alongside the upregulated expression of pulegone reductase (PR) genes and the menthol dehydrogenase (*MDH*) gene, elucidates the intricate molecular mechanisms underpinning the protective role of SA and GR24. These findings highlight the therapeutic potential of these phytohormones in enhancing plant resilience against environmental stressors and underscore their significance in promoting sustainable agricultural practices. This study paves the way for future research to optimize the use of SA and GR24 in crop improvement programs, aiming to bolster plant health and productivity in the face of increasing environmental challenges.

## Figures and Tables

**Table 1:** Physicochemical properties of the soil samples from the College of Agriculture and Natural Resources, University of Tehran farm. EC denotes electrical conductivity, and OC denotes organic carbon.

Mineral Absorbable (mg kg^−1^)				Texture Loam			OC	EC	PH
Cd	K	P	N	Silt	Clay	Sand	0.81	1.57 D Sm^−1^	8.2
0.5>	166	33.1	900	24%	21%	39%			

**Table 2:** Forward and reverse primer sequences and sizes of the PCR products.

Gene	Gene Bank (Accession Number)	Primers	Primer Size (bp)	TM
Pulegone reductase (*IPR*)	AY300163.1	F: CACAAGCCCTCATTCCTCTCT	21	60
		R: CTCTTTCCGCCGCAAAATGT	20	
Menthofuran synthase (*MFS*)	AF346833.1	F: GAGATGTTCATGGCGCTGAC	20	60
		R: CCACTTCTGCATCGACGCC	18	
Menthol Dehydrogenase (*MDH*)	KT796560.1	F: GGTAAATTGCAACAAAACAACTGG	24	60
		R: TTCAGCACCTTCAGCTTCAC	20	
*Actin*		F: GCTGGATTTGCTGGAGATGATG	22	60
		R: TCCATATCATCCCAGTTGCTGAC	23	

## Data Availability

The dataset is available upon request from the authors.
